# Comment on “Effects of Waterpipe Tobacco Smoking on the Spirometric Profile of University Students in Palestine: A Cross-Sectional Study”

**DOI:** 10.1155/2022/9831574

**Published:** 2022-01-05

**Authors:** Helmi Ben Saad

**Affiliations:** University of Sousse, Farhat HACHED Hospital, Heart Failure (LR12SP09) Research Laboratory, Sousse, Tunisia

I read with great interest the study of Nazzal et al. [1] aiming to determine the spirometric profile of young adults' exclusive narghile smokers (ENS). Such studies should be encouraged for two reasons. Firstly, ignoring the serious impacts of narghile smoking on the lungs will surely lead to a worldwide public health problem, which physicians should undertake to prevent [2]. Secondly, the 2015 World Health Organization (WHO) advisory note on narghile smoking [2] recommended further research should be conducted relating to narghile smoking-associated disease risk. However, in the paper by Nazzal et al. [1], three methodological points were noted and should be highlighted.

The first point relates to a statistical flaw. In their article, Nazzal et al. [1] did not differentiate between the “statistical” and the “clinical” significance approaches [3]. Nazzal et al. [1] have opted for the statistical significance approach with a *p* value <0.05 considered as significant. Nowadays, this approach tends to be discouraged. For example, it is true that the mean value of the forced expiratory volume in one second, expressed as a percentage of the predicted value (FEV_1_ (%)), of the ENS group was significantly lower than the one of the non-smoker group (95.9 ± 8.5 vs. 100.5 ± 9.3%, respectively, *p*=0.002); however, both reported mean values remained in the FEV_1_ normal range (i.e., ≥lower limit of normal (LLN) [4]). For that reason, it would have been better to opt for the clinical significance approach [3] and to compare the frequencies of subjects with lower spirometric parameters (e.g., <LLN [4], or *z*-score <−1.64 [5]). This clinical significance approach was previously applied in a Tunisian study [6], aiming to compare the plethysmographic parameters of ENS with age- and height-matched exclusive cigarette smokers (ECS). The authors concluded that, compared to the ECS group, the ENS one had significantly lower frequencies of subjects with reduced FEV_1_ (88 vs. 47%, respectively) or FVC (52 vs. 28%, respectively) [6]. In Nazzal et al. [1], the application of the aforementioned specific approach study would likely result in changes to some of the article's conclusions.

Point 2 is concerning the lack of precision about which spirometric predicted values were applied. To the best of this author's knowledge, no specific spirometric norms were published for the Palestinian population. In practice, the interpretation basis of spirometric parameters relies upon the comparison of the measured values with the predicted ones from a relevant healthy population with a similar ethnic background. It is important to note that some physicians accept the default settings for reference equations (e.g., European Community for Steel and Coal [7] and Global Lung Initiative 2012 [5]). Recent papers specific to some Arab populations clearly demonstrated that the use of the default “foreign” spirometric norms resulted in misinterpretation of spirometry parameters in a significant proportion of subjects [8, 9].

Point 3 relates to the lack of measurement of lung volumes. In a previous study, entitled “Spirometric Profile of Narghile Smokers” [10], it was visibly reported that 36% and 14% of ENS have, respectively, restrictive ventilatory defect and lung hyperinflation. These findings were confirmed in a case-control study [6], where the authors reported that, compared to the ECS group, the ENS one had a significantly lower frequency of subjects with higher residual volume (57 vs. 36%, respectively). Since it is important to investigate smokers' lung volumes, it would have been desirable to discuss the lack of measure of lung volumes as a methodological limitation.

In conclusion, it is clear that narghile smoking still displays many patterns related to the three following transformative phases of respiratory chronic conditions (http://www.who.int/classifications/icf/en/, last access: March 15^th^ 2021): deficiency, incapacity, and social disadvantage. This Letter to the Editor is a call to encourage future research to detect the real impact of narghile smoking on respiratory deficiency (e.g., lung function parameters). However, this future research would benefit from more solid methodological rigor.
